# Supplementary material for: Biofilm formation in the lung contributes to virulence and drug tolerance of Mycobacterium tuberculosis
Source: Nat Commun. 2021 Mar 11;12:1606. doi: 10.1038/s41467-021-21748-6 (PMC7952908; doi:10.1038/s41467-021-21748-6)
Supplement: Supplementary file 1 — Supplementary Information [file 41467_2021_21748_MOESM1_ESM.pdf]

Biofilm formation in the lung contributes to virulence and drug tolerance of *Mycobacterium tuberculosis*

Authors: Poushali Chakraborty[1], Sapna Bajeli[1], Deepak Kaushal[2], Bishan Dass Radotra[3] and Ashwani Kumar[1,4*]

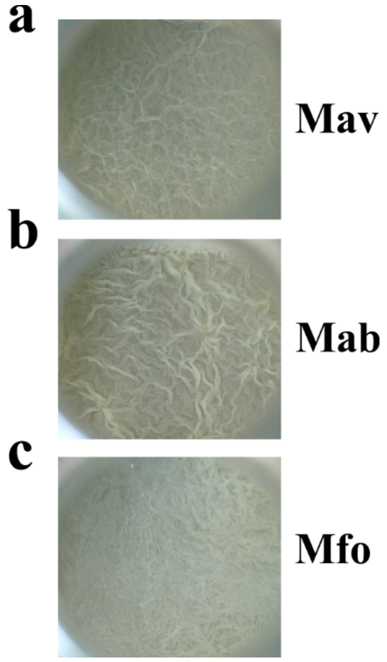

Supplementary Fig. 1| Pellicle biofilms formed by NTMs. a-c) Pellicle biofilms of Mav (**a**), Mab (**b**) and Mfo (**c**) formed as a thick mat at the air-media interface.

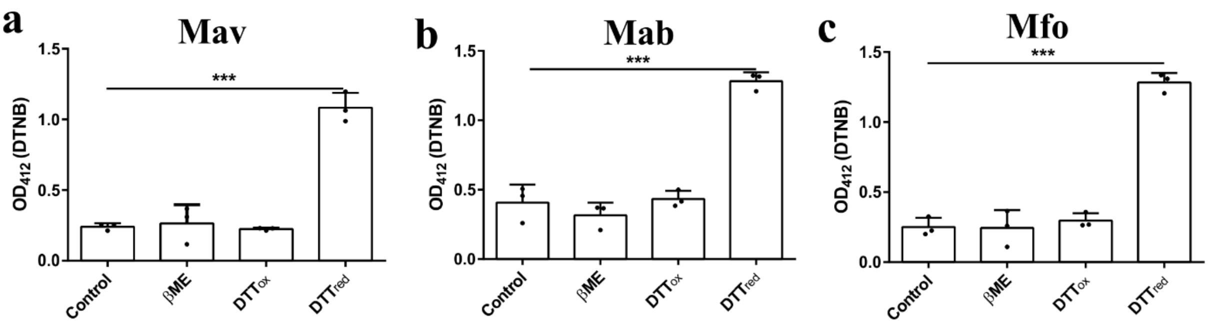

Supplementary Fig. 2| Estimation of intracellular thiol levels in NTMs. a-c) Logarithmic phase cultures of Mav (**a**), Mab (**b**) and Mfo (**c**) were exposed to 6 mM DTT and DTNB assay was performed to estimate the amount of intracellular thiol level. The column bar graphs were plotted using GraphPad Prism 6 and represented as mean (± s.e.m). Statistical significance was determined using Student's *t*- test (two tailed). For **a**, ***P = 0.0002, for **b**, ***P= 0.0005 and for **c**, ***P<0.0001). All data are representative of three independent biological experiments performed in triplicates. All source data are provided as a Source Data file.

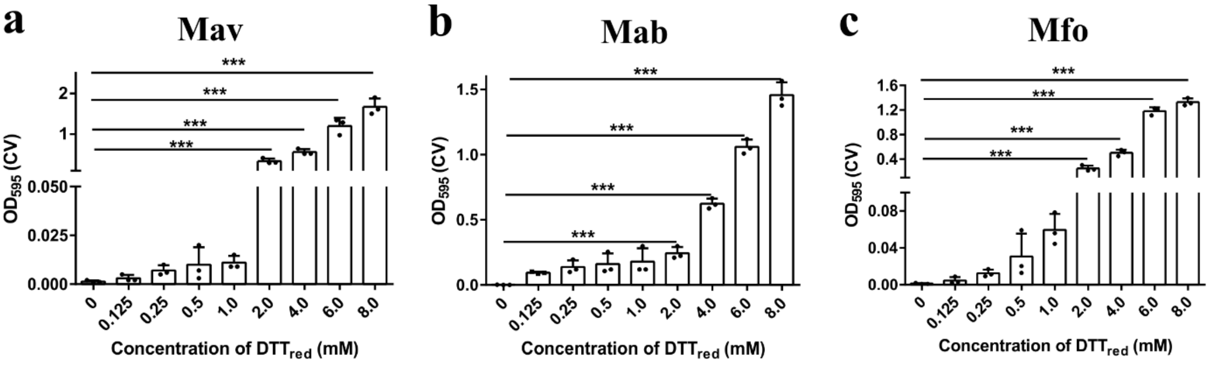

Supplementary Fig. 3| DTT requirements of NTMs to induce biofilm formation. a-c) Exponential cultures of Mav (**a**), Mab (**b**) and Mfo (**c**) were subjected to a wide range of DTT concentrations (0.125 mM to 8 mM) to determine the minimum concentration required for enhancing the biofilm formation. While Mav and Mfo requires around 2 mM, Mab can initiate biofilm formation from 0.125 mM DTT only. However it must be noted that bigger masses of bacterial communities form in response to 4 mM DTT. The column bar graphs were plotted using GraphPad Prism 6 and represented as mean (± s.e.m). Statistical significance was determined using Student's *t*- test (two tailed). For **a**, ***P= 0.0009 (column A vs column F), ***P= 0.0002 (column A vs column G), ***P= 0.0005 (column A vs column H), ***P= 0.0001 (column A vs column I), for **b**, ***P= 0.0009 (column A vs column F), ***P<0.0001 (column A vs column G), ***P<0.0001 ((column A vs column H), ***P<0.0001 (column A vs column I) and for **c**, ***P= 0.0008 (column A vs column F), ***P<0.0001 (column A vs column G), ***P<0.0001 ((column A vs column H), ***P<0.0001 (column A vs column I). All data are representative of three independent biological experiments performed in triplicates. All source data are provided as a Source Data file.

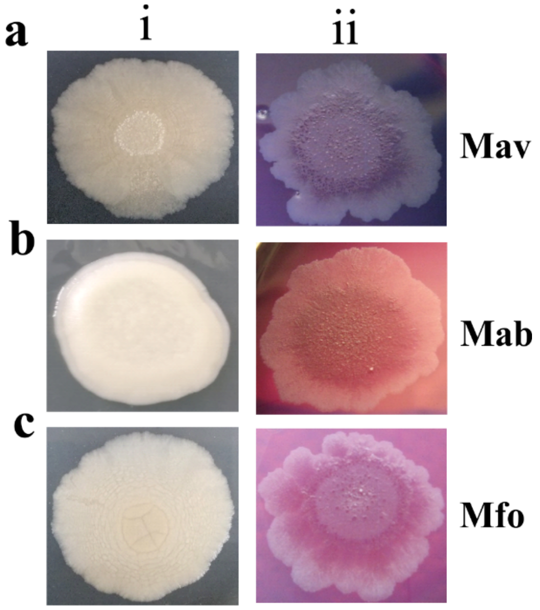

Supplementary Fig. 4| Macrocolony biofilms of NTMs. a-c) Macrocolonies of Mav (**a**), Mab (**b**) and Mfo (**c**) either on 7H11 plate (**i**) or 7H11 plate supplemented with CR and CBB (**ii**).

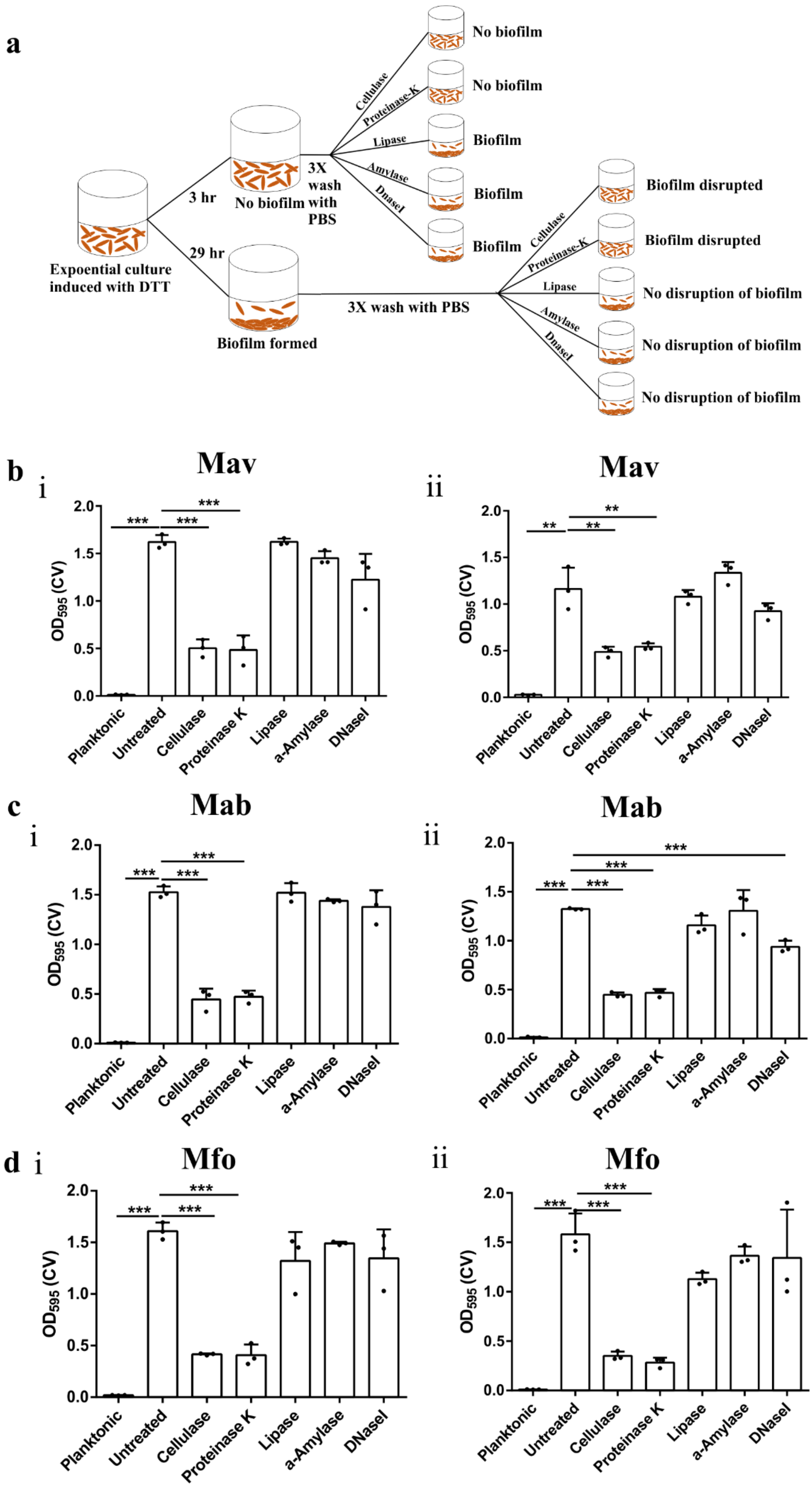
a
No biofilm
No biofilm
Biofilm
Biofilm
Biofilm
Cellulase
Proteinase-K
Lipase
Amylase
DnaseI
3X wash
No biofilm with PBS
3 hr
29 hr
Expoential culture induced with DTT
Biofilm formed
3X wash with PBS
Biofilm disrupted
Biofilm disrupted
No disruption of biofilm
No disruption of biofilm
No disruption of biofilm
b
Mav
Mav
c
Mab
Mab
d
Mfo
Mfo
i
ii
OD595 (CV)
Planktonic
Untreated
Cellulase
Proteinase K
Lipase
a-Amylase
DNaseI

Supplementary Fig. 5| Effects of different polymer degrading enzymes on the integrity of NTM biofilms. a) Cartoon representation of the experimental procedure. **b-d)** DTT treated exponential phase cultures of Mav (**a**), Mab (**b**) and Mfo (**c**) were exposed to Cellulase, Proteinase K, Lipase, α-Amylase and DNaseI after 29 hours of DTT treatment (**i**) i.e., after the formation of biofilms or after 3 hours of DTT treatment (**ii**). Biofilm formation was fully compromised when treated with Cellulase and Proteinase K, and partially when treated with DNaseI. Biofilms were disintegrated upon treatment of with Cellulase and Proteinase K. The column bar graphs were plotted using GraphPad Prism 6 and represented as mean (± s.e.m). Statistical significance was determined using Student's *t*- test (two tailed). For **a (i),** ***P<0.0001 (column A vs column B), ***P<0.0001 (column B vs column C), ***P= 0.0003 (column B vs column D), for **a (ii)**, **P= 0010, **P= 0.0077 (column B and column C) and **P= 0.0098 (column B and column D). For **b (i),** ***P<0.0001 (column A vs column B), ***P= 0.0001 (column B vs column C), ***P<0.0001 (column B vs column D), for **b (ii)**, ***P<0.0001, ***P<0.0001 (column B and column C), ***P<0.0001 (column B and column D) and ***P= 0.0004 (column B and column G). For **c (i),** ***P<0.0001 (column A vs column B), ***P<0.0001 (column B vs column C), ***P= 0.0003 (column B vs column D), for **c (ii)**, ***P= 0.0002 (column A vs column B), ***P= 0.0006 (column B and column C) and ***P= 0.0005 (column B and column D). All data are representative of three independent biological experiments performed in triplicates. All source data are provided as a Source Data file.

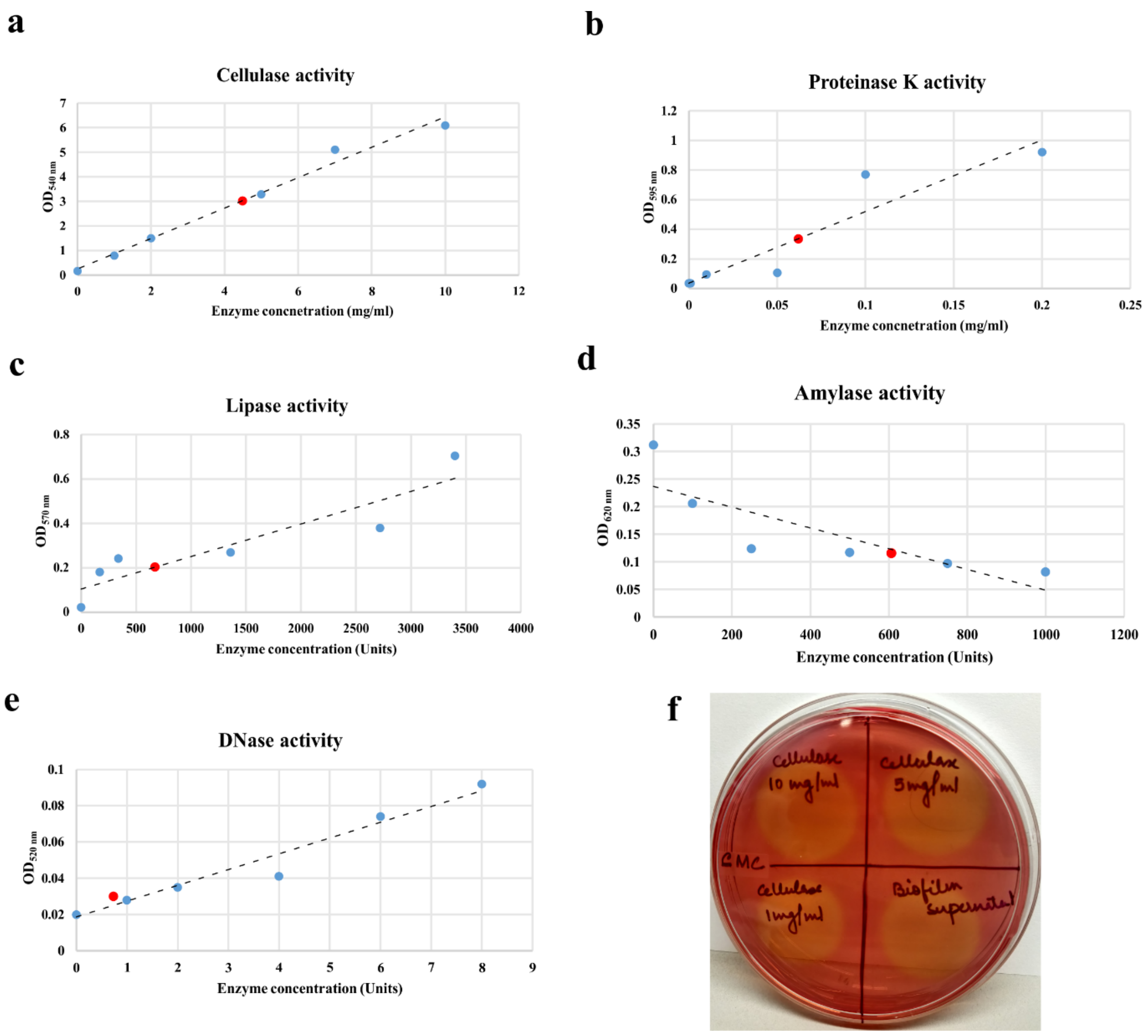

Supplementary Fig. 6| Estimation of enzyme activities in biofilm supernatant. a-e) NTM biofilms were treated with Cellulase, Proteinase K, Lipase, α-Amylase and DNaseI after 29 hours of DTT treatment, after respective hours of enzyme treatment, the biofilm supernatants were isolated and checked for the respective enzyme activity. Standard curves were plotted for all the enzymes and Cellulase (**a**), Proteinase K (**b**), Lipase (**c**), α Amylase (**d**) and DNaseI (**e**) activities were checked in the biofilm supernatant. Red dot indicates the biofilm supernatant, blue dots correspond to standards. **f)** Activity of cellulase and biofilm supernatant shown on CMC agar plates, stained with CR and washed with 1M NaCl. The clear zone shows the digested cellulose.

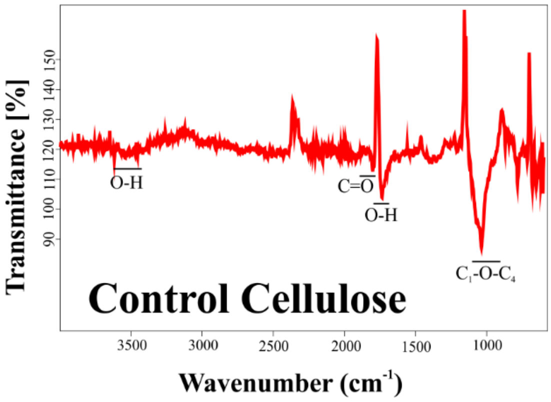

Supplementary Fig. 7| Characterization of biofilm matrix polysaccharides. Commercially available, anhydrous cellulose was dissolved in TFA and subjected to FTIR.

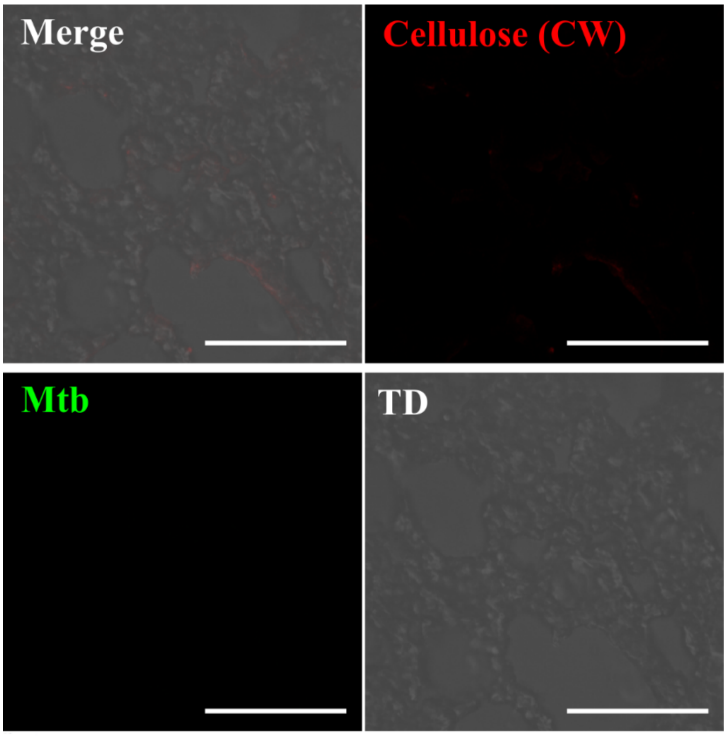

Supplementary Fig. 8| Staining of Mtb infected mice lungs with CW. Mtb infected mice lung sections were stained with CW for cellulose and Auramine O – Rhodamine B for Mtb, and fields lacking the bacilli did not stain for CW. All data are representative of three independent biological experiments. Scale bars correspond to 50 µm.

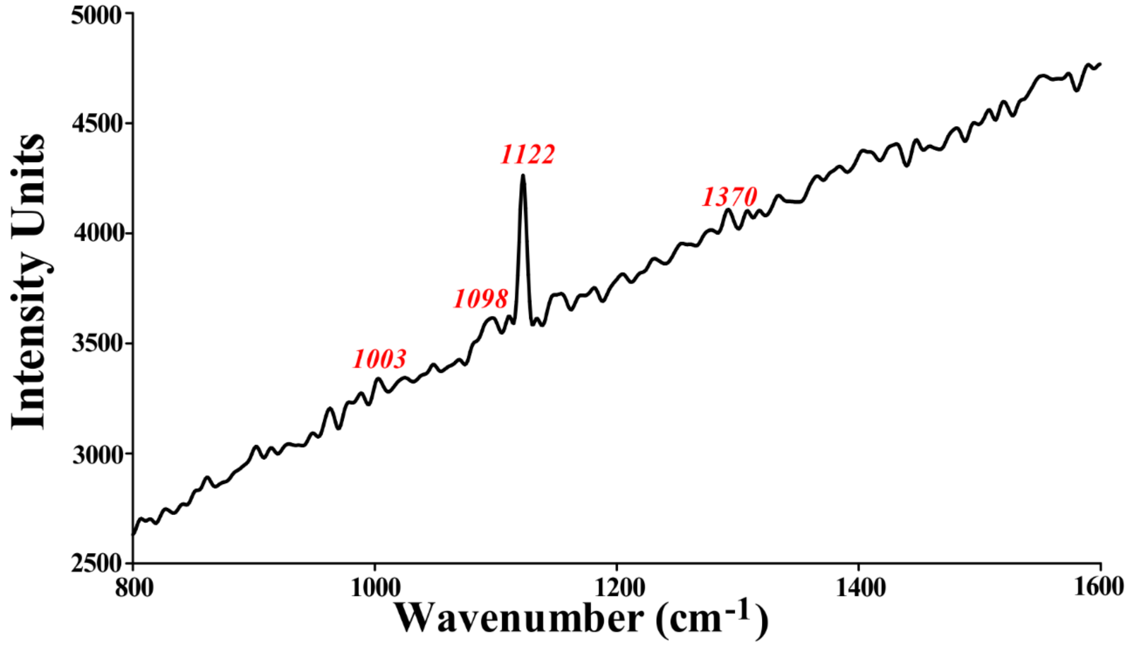

Supplementary Fig. 9| Raman microscopy profile of crystalline cellulose. Commercially available anhydrous cellulose was placed on a glass slide and analyzed using a Raman microscope.

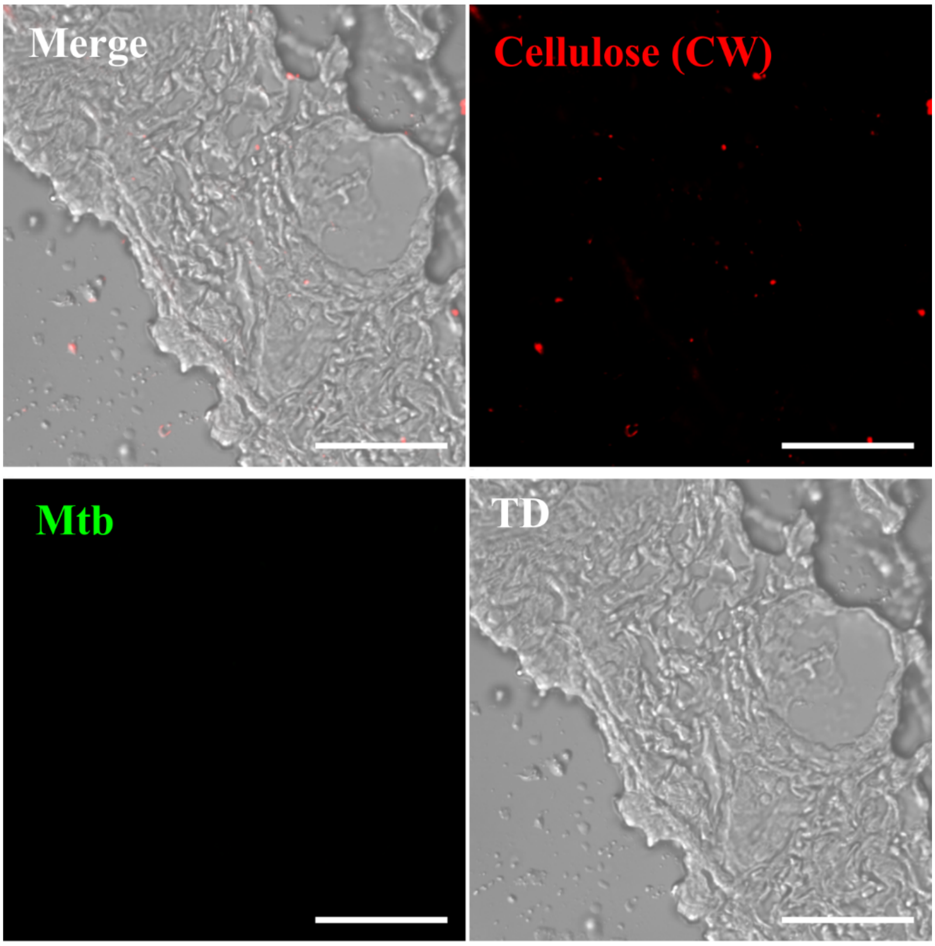

Supplementary Fig. 10| Staining of Mtb infected macaque lungs with CW. Mtb infected macaque lung sections were stained with CW for cellulose and Auramine O – Rhodamine B for Mtb, and fields lacking the bacilli did not stain for CW. All data are representative of three independent biological experiments. Scale bars correspond to 50 μm.

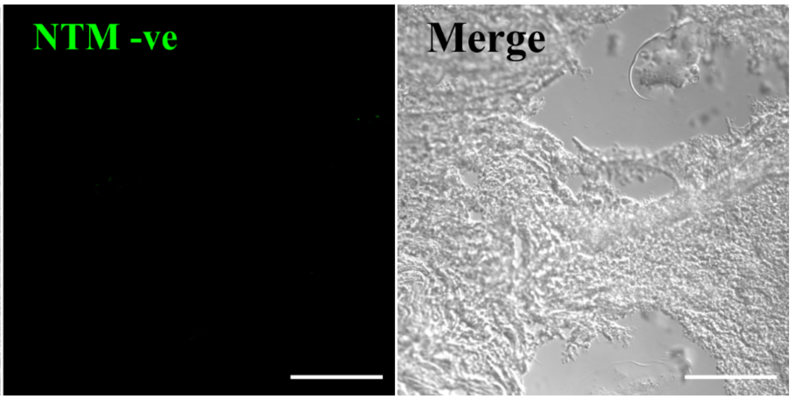

Supplementary Fig. 11| FISH with NTM specific 16S primers was utilized for identifying lung tissues infected with Mtb. The Mtb infected lung sections did not stain for NTM specific probes. All data are representative of three independent biological experiments. Scale bars correspond to 50 μm.

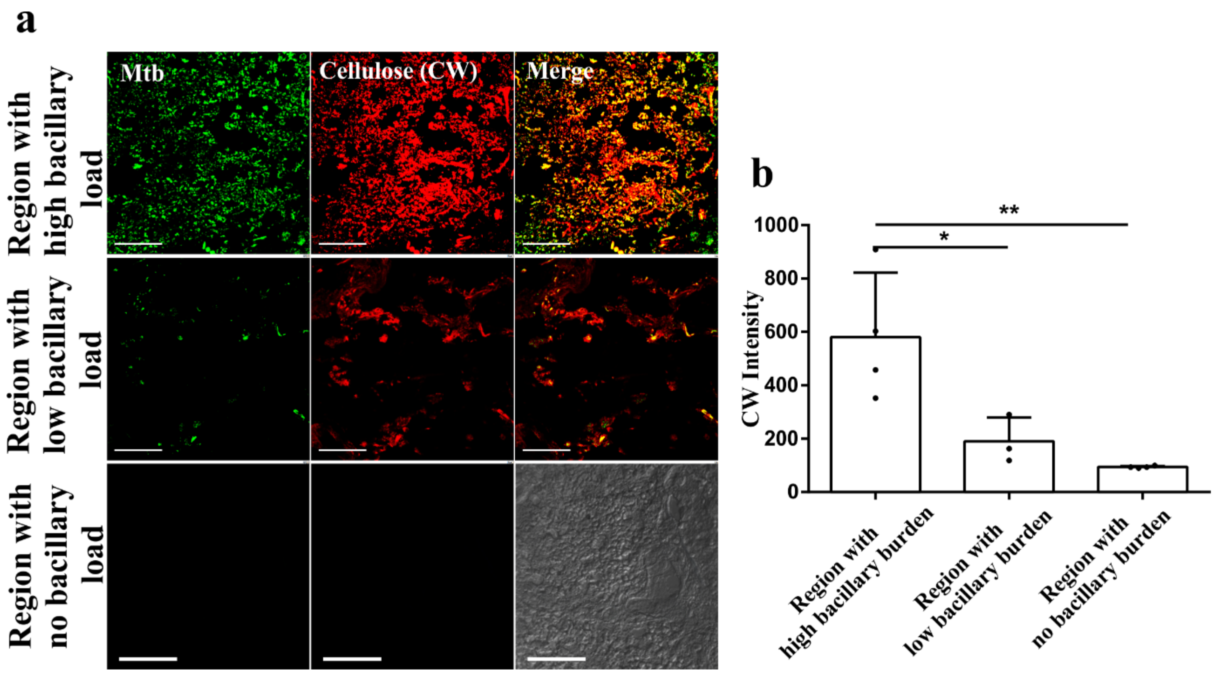

Supplementary Fig. 12| CW staining corresponds to bacterial load. a) CW staining of loci with high bacterial load, moderate or low bacterial load and loci wherein Mtb cells were absent. **b**) Quantitation of CW florescence intensity using NIS elements and the column bar graph plotted on GraphPad Prism 6 software and represented as mean (± s.e.m). Statistical significance was determined using Student's *t*- test (two tailed). For **b**, **P*= 0.0475 and ***P*= 0.0069. Scale bars correspond to 50 μm. All source data are provided as a Source Data file.

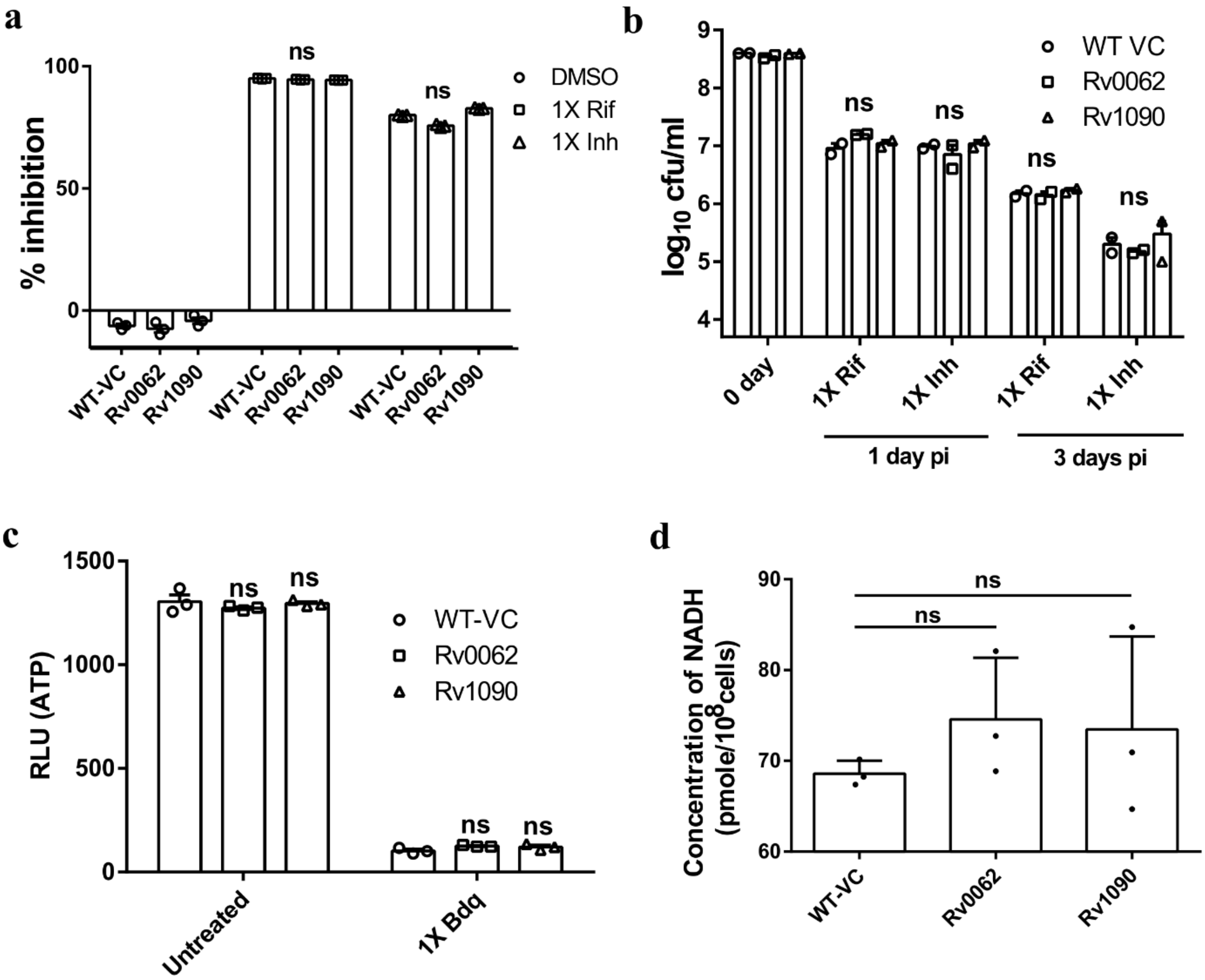

Supplementary Fig. 13| Effect of antimycobacterials on engineered Mtb strains. a) In order to examine sensitivity of the engineered strains towards Rif and INH, Alamar Blue assay of WT-VC, Rv0062 and Rv1090 was performed and % inhibition was checked for each of the strains with respect to Rif and INH. (Circle represents DMSO, Square represents Rif and Triangle represents INH). **b)** Sensitivity of the engineered strains towards Rif and INH was also evaluated by CFU of WT-VC, Rv0062 and Rv1090 in presence of 1X Rif and 1X INH for 1 day and 3 days post treatment. From the graphs it is clearly evident that all the three strains behave similarly in response to the drugs. (Circle represents WT-VC, Square represents Rv0062 and Triangle represents Rv1090). **c)** ATP levels were enumerated for WT-VC, Rv0062 and Rv1090 with or with Bedaquiline, which showed similar levels in all the three strains. (Circle represents WT-VC, Square represents Rv0062 and Triangle represents Rv1090). **d)** NADH levels were estimated for WT-VC, Rv0062 and Rv1090 which showed equal levels for all the three strains. The bar graphs were plotted using GraphPad Prism 6 and represented as mean (± s.e.m). Statistical significance was determined using two way ANOVA for (**a**), (**b**) and (**c**) and Student's *t*- test (two tailed) for (**d**). All data are representative of two (for (**b**)) and three (for (**a**), (**c**) and (**d**)) independent biological experiments performed in triplicates unless otherwise mentioned. All source data are provided as a Source Data file.
