# Supplementary material for: Biofilm formation in the lung contributes to virulence and drug tolerance of Mycobacterium tuberculosis
Source: Nat Commun. 2021 Mar 11;12:1606. doi: 10.1038/s41467-021-21748-6 (PMC7952908; doi:10.1038/s41467-021-21748-6)
Supplement: Supplementary file 3 — Reporting Summary [file 41467_2021_21748_MOESM3_ESM.pdf]

nature research

Corresponding author(s): Ashwani Kumar

Last updated by author(s): Jan 31, 2021

Reporting Summary

Nature Research wishes to improve the reproducibility of the work that we publish. This form provides structure for consistency and transparency in reporting. For further information on Nature Research policies, see our Editorial Policies and the Editorial Policy Checklist.

Statistics

For all statistical analyses, confirm that the following items are present in the figure legend, table legend, main text, or Methods section.

n/a	Confirmed	
☐	☒	The exact sample size (n) for each experimental group/condition, given as a discrete number and unit of measurement
☐	☒	A statement on whether measurements were taken from distinct samples or whether the same sample was measured repeatedly
☐	☒	The statistical test(s) used AND whether they are one- or two-sided<br>*Only common tests should be described solely by name; describe more complex techniques in the Methods section.*
☒	☐	A description of all covariates tested
☒	☐	A description of any assumptions or corrections, such as tests of normality and adjustment for multiple comparisons
☐	☒	A full description of the statistical parameters including central tendency (e.g. means) or other basic estimates (e.g. regression coefficient) AND variation (e.g. standard deviation) or associated estimates of uncertainty (e.g. confidence intervals)
☐	☒	For null hypothesis testing, the test statistic (e.g. F, t, r) with confidence intervals, effect sizes, degrees of freedom and P value noted<br>*Give P values as exact values whenever suitable.*
☒	☐	For Bayesian analysis, information on the choice of priors and Markov chain Monte Carlo settings
☒	☐	For hierarchical and complex designs, identification of the appropriate level for tests and full reporting of outcomes
☒	☐	Estimates of effect sizes (e.g. Cohen's d, Pearson's r), indicating how they were calculated

Our web collection on statistics for biologists contains articles on many of the points above.

Software and code

Policy information about availability of computer code

Data collection	Softwares used to collect data were NIS elements, Agilent spectrophotometer, Renishaw Raman software.
Data analysis	Softwares used to analyze data GraphPad Prism 6, ImageJ and NIS elements

For manuscripts utilizing custom algorithms or software that are central to the research but not yet described in published literature, software must be made available to editors and reviewers. We strongly encourage code deposition in a community repository (e.g. GitHub). See the Nature Research guidelines for submitting code & software for further information.

Data

Policy information about availability of data

All manuscripts must include a data availability statement. This statement should provide the following information, where applicable:

- Accession codes, unique identifiers, or web links for publicly available datasets
- A list of figures that have associated raw data
- A description of any restrictions on data availability

All the relevant data are presented in the manuscript and supplemetary information. Also a Source Data file has been attached along with the main manuscript.

Field-specific reporting

Life sciences study design

All studies must disclose on these points even when the disclosure is negative.

Sample size	The sample size for each experiment has been mentioned in the figure legends of the relevant figures. A minimum of 3 biological replicates have been taken for each experimental set along with 3 technical replicates, unless otherwise mentioned.
Data exclusions	No data exclusions
Replication	Reproducibility of the experiments was determined through biological and technical replicates wherever required. This is specifically mentioned in the figure legends. Also reproducibility was determined using statistical analysis.
Randomization	No randomization method was used.
Blinding	Data collection and analysis were not performed blind.

Reporting for specific materials, systems and methods

We require information from authors about some types of materials, experimental systems and methods used in many studies. Here, indicate whether each material, system or method listed is relevant to your study. If you are not sure if a list item applies to your research, read the appropriate section before selecting a response.

Materials & experimental systems

n/a	Involved in the study	
☐	☒	Antibodies
☒	☐	Eukaryotic cell lines
☒	☐	Palaeontology and archaeology
☐	☒	Animals and other organisms
☐	☒	Human research participants
☒	☐	Clinical data
☒	☐	Dual use research of concern

Methods

n/a	Involved in the study	
☒	☐	ChIP-seq
☒	☐	Flow cytometry
☒	☐	MRI-based neuroimaging

Antibodies

Antibodies used	Rabbit pAb to S tag [ab18588, Abcam, Lot- GR174220-1], mouse anti-rabbit IgG-HRP [sc-2357, Santa Cruz Biotechnology, Lot-E1618]
Validation	Relevant citation: Ravindran MS, Bagchi P, Inoue T, Tsai B (2015) A non-enveloped virus hijacks host disaggregation machinery to translocate across the endoplasmic reticulum membrane. PLoS Pathog 11(8): e1005086. doi: 10.1371/journal.ppat.1005086

Animals and other organisms

Policy information about studies involving animals; ARRIVE guidelines recommended for reporting animal research

Laboratory animals	8-12 weeks old female C57BL/6J mice were used in the study. They were housed with 12 hours of light and dark cycles, with 23C temperature and 50 % humidity. Tissue samples of rhesus macaques were obtained from Dr. Deepak Kaushal . All these experiments were performed according to ARRIVE guidelines.
Wild animals	No wild animals were used in the study.
Field-collected samples	No field collected samples were used in the study.
Ethics oversight	All experiments pertaining to mice were approved b y the Institutional Animal Ethics Committee of CSIR-IMTech (IAEC/17/27). All the mice experiments were performed according to the guidelines issued by the Committee for the Purpose of Control and Supervision of Experiments on Animals (No. 55/1999/CPCSEA) under the Prevention of Cruelty to Animals Act 1960 and amendments introduced in 1982 by Ministry of Environment and Forest, Govt. of India.

Note that full information on the approval of the study protocol must also be provided in the manuscript.

Human research participants

Policy information about studies involving human research participants

Population characteristics	Paraffin embedded lung sections of human cadavers were obtained from Dr. Bishan D. Radotra. The cadavers were irrespective of age and gender. They were either suffering from tuberculosis or not from tuberculosis. Detailed information has been mentioned in the methods section of the manuscript.

Recruitment	The lung section from the cadavers were chosen randomly based on whether they were suffering or not from tuberculosis.
Ethics oversight	All experiments pertaining to human samples were approved by the Institutional Ethics Committee of CSIR-IMTech [IEC(Sept 18) #5]

Note that full information on the approval of the study protocol must also be provided in the manuscript.
